# Nitration of Chitin Monomer: From Glucosamine to Energetic Compound

**DOI:** 10.3390/molecules26247531

**Published:** 2021-12-12

**Authors:** Hui Dou, Yuxuan Zheng, Manyi Qu, Peng Chen, Chunlin He, Michael Gozin, Siping Pang

**Affiliations:** 1 Experimental Center of Advanced Materials, School of Materials Science and Engineering, Beijing Institute of Technology, Beijing 100081, China; douhui@bit.edu.cn (H.D.); pengchen@bit.edu.cn (P.C.); 2 State Key Laboratory of Explosion Science and Technology, Beijing Institute of Technology, Beijing 100081, China; yxzheng@bit.edu.cn; 3 Shanxi North Xingan Chemical Industry Co., Ltd., Taiyuan 030008, China; qumanyi2005@163.com; 4 Yangtze Delta Region Academy, Beijing Institute of Technology, Jiaxing 314019, China; 5 Chongqing Innovation Center, Beijing Institute of Technology, Chongqing 401120, China; 6 School of Chemistry, Faculty of Exact Sciences, Tel Aviv University, Tel Aviv 6997801, Israel; 7 Center for Nanoscience and Nanotechnology, Tel Aviv University, Tel Aviv 6998701, Israel; 8 Center for Advanced Combustion Science, Tel Aviv University, Tel Aviv 6997801, Israel

**Keywords:** energetic materials, *N*-acetyl-d-glucosamine, nitration, propellants

## Abstract

The nitration of chitin monomer in a mixture of nitric acid and acetic anhydride was conducted and a highly nitrated (3R,4R,6R)-3-acetamido-6-((nitrooxy)methyl)tetrahydro-2*H*-pyran-2,4,5-triyl trinitrate (**1**) was obtained. Its structure was fully characterized using infrared spectroscopy, NMR spectroscopy, elemental analysis, and X-ray diffraction. Compound **1** possesses good density (ρ: 1.721 g·cm^−3^) and has comparable detonation performance (V_d_: 7717 m·s^−1^; P: 25.6 GPa) to that of nitrocellulose (NC: V_d_: 7456 m·s^−1^; P: 23 GPa; I_sp_ = 239 s) and microcrystalline nitrocellulose (MCNC; V_d_: 7683 m·s^−1^; P: 25 GPa; I_sp_ = 250 s). However, Compound **1** has much lower impact sensitivity (IS: 15 J) than the regular nitrocellulose (NC; IS: 3.2 J) and MCNC (IS: 2.8 J). Compound **1** was calculated to exhibit a good specific impulse (I_sp_: 240 s), which is comparable with NC (I_sp_: 239 s) and MCNC (I_sp_: 250 s). By replacing the nitrocellulose with Compound **1** in typical propellants JA2, M30, and M9, the specific impulse was improved by up to 4 s. These promising properties indicate that Compound **1** has a significant potential as an energetic component in solid propellants.

## 1. Introduction

In recent years, with the scarcity of non-renewable resources, such as oil and coal, and with the severe environmental pollution problems that they cause, green, renewable and environment-friendly materials have attracted increasing attention [1,2,3,4,5]. Chitin biopolymer (C_8_H_13_NO_5_)_n_ is one of the most abundant of the polysaccharides in nature. It can be found in the shells of crustaceans, such as shrimps and crabs, as well as in various insects, and in the cell walls of fungi [6,7,8,9]. Chitin is a promising raw material for various applications due to its abundance, low price, and chemical and biological properties [10]. Furthermore, its biocompatibility, biodegradability, strong antibacterial effect, and lack of toxicity means that chitin can be used in a broad range of biomedical applications, in the food industry, and in agriculture [11,12,13,14,15]. The chemical structure of chitin is somewhat similar to cellulose. The monomer of chitin, *N*-acetyl-d-glucosamine, can be readily obtained through the hydrolysis of chitin [16,17]. *N*-acetyl-d-glucosamine was reported to show promising biological activity and it is also widely used in the pharmaceutical, food, biomedical, and chemical industries [18,19,20].

**Figure 1:**
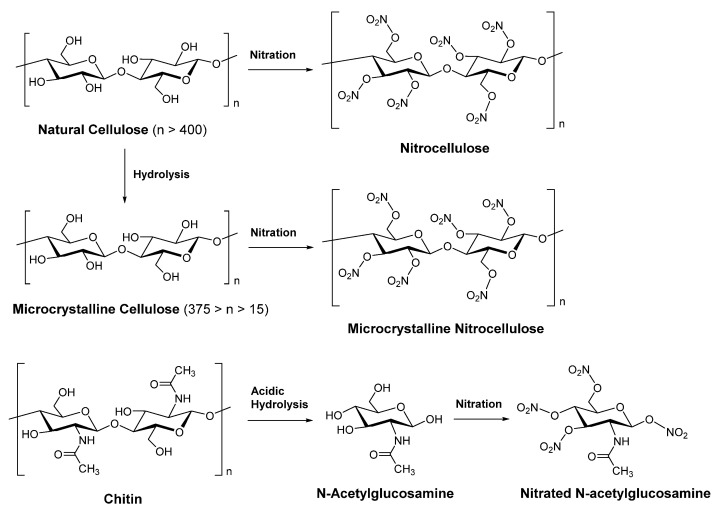
Synthesis of microcrystalline nitrocellulose, preparation of *N*-acetylglucosamine and nitrated *N*-acetyl-glucosamine.

The nitration of cellulose results in the formation of nitrocellulose (NC), which is one of the most commonly used energetic polymers in defense and civilian applications [21,22,23,24]. Nitrocellulose has some limitations, including hazardous sensitivity to mechanical impacts and inhomogeneity. At present, the research on the desensitization of nitrocellulose focuses on structural modifications and the use of nanotechnology [25,26,27]. The research related to the use of nano-nitrocellulose shows that the mechanical sensitivity of the latter material is favorably reduced. However, the cost of nano-nitrocellulose is significantly higher than the cost of ordinary nitrocellulose. The nitration of chitosan has also been reported recently, and it shows great potential as a solid propellant candidate [28]. To the best of our knowledge, no reports were published regarding the nitration of *N*-acetyl-d-glucosamine (Figure 1). Our continuous research interests in searching for novel energetic compounds with accessible starting materials, low costs, and promising properties motivates us to study the nitration of *N*-acetyl-d-glucosamine. The nitration was conducted using a mixture of acetic anhydride and nitric acid, and the product was fully characterized through single-crystal X-ray diffraction analysis, elemental analysis, differential scanning calorimetry (DSC), nuclear magnetic characterization, and IR spectroscopy. In addition, its energetic properties were analyzed experimentally and theoretically, and compared with those of conventional NC and emerging microcrystalline nitrocellulose (MCNC). Its comparable detonation properties and similar structure to that of nitrocellulose reveal its potential as a solid propellant.

## 2. Results and Discussion

### 2.1. Synthesis

**Scheme 1:**
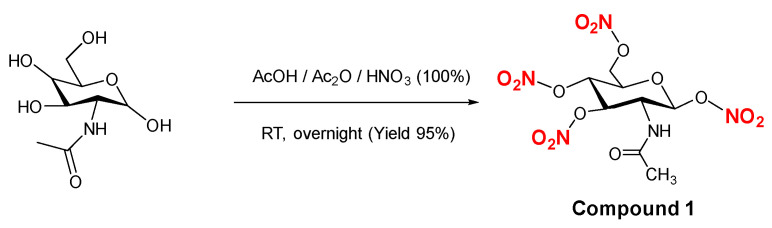
The synthetic route of Compound **1** production.

A compound, (3R,4R,6R)-3-acetamido-6-((nitrooxy)methyl)tetrahydro-2H-pyran-2,4,5-triyl trinitrate (**1**) was prepared with a 95% yield by nitrating *N*-acetyl-d-glucosamine with a mixture of acetic acid, acetic anhydride, and nitric acid at room temperature (Scheme 1). After the reaction was completed, the above mixture was poured onto ice water, and the precipitate was collected via filtration and dried under vacuum conditions to produce Compound **1**, which is a fine, white solid that is pure enough to pass the elemental analyses.

### 2.2. Single-Crystal X-ray Analysis

**Figure 2:**
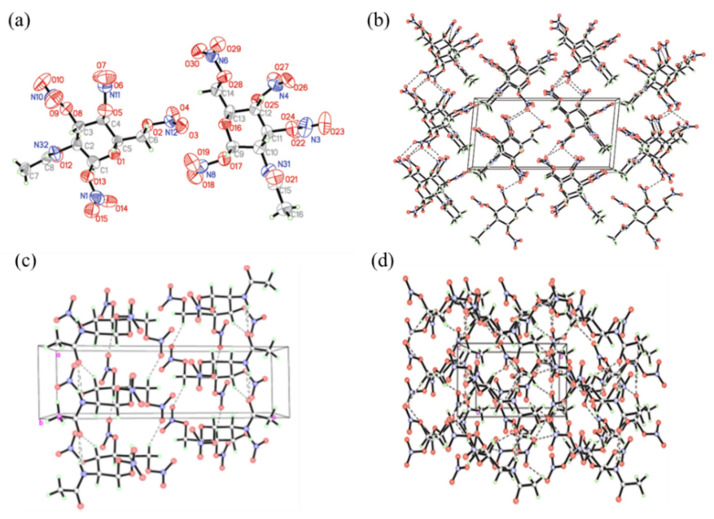
(**a**) Crystal structure of Compound **1**; (**b**) a-axis crystal stacking diagram; (**c**) b-axis crystal stacking diagram; (**d**) c-axis crystal stacking diagram.

Crystals of Compound **1** suitable for single-crystal X-ray diffraction were obtained by dissolving this compound in a minimum amount of acetonitrile and allowing the solvent to evaporate slowly. The unit cell of Compound **1**, which crystallizes in the triclinic space group P_1_, contains two moieties, with a good crystal density of 1.722 g·cm*^−^*^3^ at 296 K. The skeleton of Compound **1** is a twisted, six-membered ring, which makes the crystal stack slightly disordered. However, from the a-axis direction, every molecule has its parallel molecules, which show orderly cross-stacking (Figure 2b). As can be seen in Figure 1, there is an extensive network of intermolecular hydrogen bonds, which is one of the main reasons for the relatively high density of Compound **1** crystals.

## 3. Physicochemical and Energetic Properties

In order to evaluate the properties of Compound **1** as energetic materials, important physical properties, such as thermal stability, density, heat of formation, detonation properties, and mechanical sensitivity were tested, and the results are shown in Table 1.

### 3.1. Density and Heat of Formation

Density is an important parameter for evaluating the detonation properties of energetic materials. The density of Compound **1** was measured using a helium pycnometer at 25 °C. The compound showed a good density of 1.721 g·cm*^−^*^3^, which is higher than that of commonly used propellant components, such as NC (1.673 g·cm*^−^*^3^) and MCNC (1.691 g·cm*^−^*^3^). This may be due to the existence of extensive intermolecular hydrogen bonds. Heat of formation (HOF) is another important parameter for evaluating the detonation performance of energetic materials. The heat of formation was calculated based on Gaussian 09 (details are provided in the Supplementary Materials). As shown in Table 1, the heat of formation of Compound **1** is −785.8 kJ·mol*^−^*^1^, lower than that of NC (−714 kJ·mol*^−^*^1^) and MCNC (−573 kJ·mol*^−^*^1^).

### 3.2. Detonation Performance

Based on the measured densities and the calculated heat of formation (HOF), the detonation velocities (V_d_) and detonation pressures (P) of this new energetic material were obtained by using the EXPLO5 (v6.05) program [30]. The detonation velocity of 7717 m·s^−1^ and a detonation pressure of 25.6 GPa for Compound **1** is comparable to that of MCNC (V_d_: 7683 m·s^−1^; P, 25 GPa). Additionally, with a calculated specific impulse of 240 s, it is a prospective candidate for a solid propellant.

In order to evaluate the potential of Compound **1** to be used as a component in various propellants, the formulations of F1, F2, and F3 were designed by replacing the same weight percentage of nitrocellulose with Compound **1** in typical nitrocellulose-based propellant formulations, including JA2, M30, and M9 [31]. Their specific impulses (I_sp_) were calculated using EXPLO5 (V 6.05) and the results are listed in Table 2. As we can see from these results, after replacing nitrocellulose with Compound **1**, the I_sp_ values of F1, F2, and F3 were increased by up to 4 s compared to that of JA2, M30, and M9, respectively, indicating the great potential for Compound **1** to act as an energetic component for advanced future propellants.

### 3.3. Hirshfeld Surface

**Figure 3:**
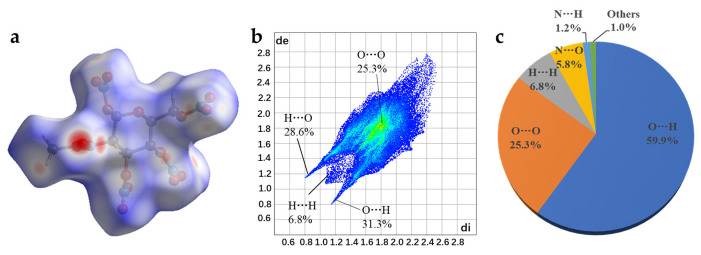
(**a**) Hirshfeld surface for Compound **1**; (**b**) fingerprint plot for Compound **1**; (**c**) individual atomic contact, percentage contribution to the Hirshfeld surface.

The Hirshfeld surfaces clearly display the intermolecular or intramolecular interactions in the crystal stacking [32]. Figure 2b shows the two-dimensional (2D) fingerprint plots; in this case, the spikes for Compound **1** are broad, suggesting that a high percentage of hydrogen bonds are present. This impression is based on the crystal structures, in which O···H and N···H have 61.1% of total contacts for Compound **1** (Figure 3c), which is higher than most energetic compounds.

### 3.4. Thermal Stability and Mechanical Sensitivity

The decomposition temperature of Compound **1** was determined using differential scanning calorimetry (DSC) at a heating rate of 10 *°*C·min^−1^. A sharp exothermic peak, corresponding to the decomposition of Compound **1**, was measured at 154 *°*C (onset temperature) (Table 1).

The sensitivity toward mechanical impact and friction were determined by standard BAM methods (Table 1). Compound **1** displayed significantly lower sensitivity to impact (15 J) than NC (3.2 J) and MCNC (2.8 J). Such relatively low sensitivity to impact could not be explained only by the Compound **1** crystal’s stacking arrangement. It is also closely related to the intramolecular and intermolecular hydrogen bonds in this crystal, as could be seen in the Hirshfeld surface of Compound **1**, which indicates the presence of a significant number of hydrogen bonds (Figure 3).

### 3.5. Electrostatic Potential

**Figure 4:**
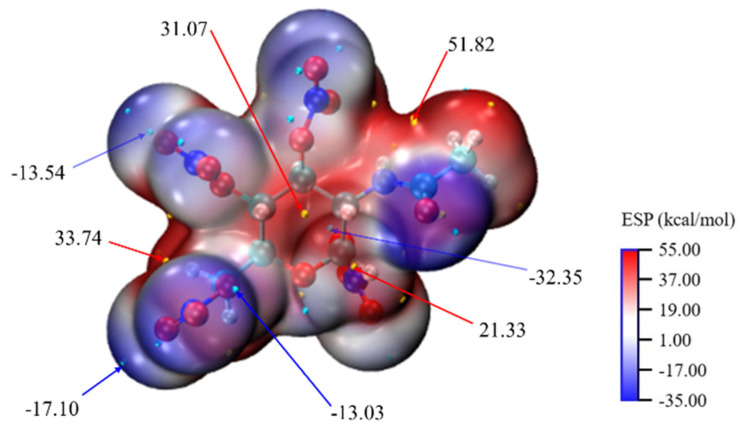
ESP-mapped molecular vdW surface of Compound **1**. The minimum and maximum values of ESP are marked as blue and red points, respectively.

The calculated electrostatic potential (ESP) is a good tool to explain the changes in molecular stability. The ESP plots of Compound **1** were evaluated by using Multiwfn (Figure 4). In energetic compounds, the sensitivities are closely related to their surface ESPs. The larger electropositive areas and higher ESP values often result in high sensitivities [32,33]. Compound **1** exhibits lower ESP maximum values, especially in *N*-acetylamino group areas (+51.82 kcal·mol*^−^*^1^). Therefore, this is one of the reasons why Compound **1** has low impact sensitivity.

## 4. Experimental Section

### 4.1. General Information

All reagents were purchased in the market and used within the specified date. Reagents were purchased from Alfa Aesar at an analytical grade and were used as received. The spectra for ^1^H and ^13^C NMR were measured on a 400 MHz (Bruker Ascend TM 400) Nuclear Magnetic Resonance spectrometer. The thermal decomposition profiles were obtained using a Differential Scanning Calorimeter (TA Instruments Company, Model: DSC25) at a scan rate of 10 °C·min^−1^. IR spectra were measured on FT-IR spectrometer (Nicolette iS50). Density was measured at room temperature on a Micromeritics AccuPyc II 1345 gas pycnometer. Elemental analyses (C, H, N) were determined using a FLASH 2000 Elementar Analyser. The impact and friction sensitivities were tested on a standard BAM drop hammer and BAM friction testers.

### 4.2. Synthesis of Compound ***1***

To a solution of N-Acetyl-D-Glucosamine (0.33 g, 1.5 mmol in a mixture of acetic acid (2.5 mL) and acetic anhydride (5 mL)), fuming nitric acid (5 mL) was added dropwise at −5 °C. After stirring for 30 min at −5 °C, the reaction mixture was allowed to warm up to room temperature and stirred overnight. Then, the reaction mixture was poured into 40 mL of ice water. The white precipitate was collected via filtration, washed with ice water (5 mL × 4) and dried under vacuum to produce Compound **1** at a 95% yield (0.57 g). Decomposition temperature (onset value: at 10 °C·min^−1^ heating rate), T_d_ = 154 °C; ^1^H NMR (CD_3_CN): δ 6.76 (s, H), 6.25 (s, H), 5.59 (s, H), 5.46 (s, H), 4.79 (s, 2H), 4.69 (s, H), 4.49 (s, H), 1.88 (s, 3H ); ^13^C NMR (CD_3_CN): δ 171.37, 97.90, 78.75, 75.35,70.51, 69.03, 49.56, 22.76 ppm; IR (cm^−1^) ṽ = 3297, 2991, 1651, 1638, 1533, 1283, 1258, 1155, 1133, 1022, 953, 890, 824, 749, 677, 638, 615, 597, 532, 493, 471; elemental analysis for C_8_H_11_N_5_O_14_ (401.20): calcd C 23.95, H 2.76, N 17.46%; found: C 24.04, H 2.72, N 17.38%.

To a solution of N-Acetyl-D-Glucosamine (0.33 g, 1.5 mmol in a mixture of acetic acid (2.5 mL) and acetic anhydride (5 mL)), fuming nitric acid (5 mL) was added dropwise at −5 °C. After stirring for 30 min at −5 °C, the reaction mixture was allowed to warm up to room temperature and stirred overnight. Then, the reaction mixture was poured into 40 mL of ice water. The white precipitate was collected via filtration, washed with ice water (5 mL × 4) and dried under vacuum to produce Compound **1** at a 95% yield (0.57 g). Decomposition temperature (onset value: at 10 °C·min^−1^ heating rate), T_d_ = 154 °C; ^1^H NMR (CD_3_CN): δ 6.76 (s, H), 6.25 (s, H), 5.59 (s, H), 5.46 (s, H), 4.79 (s, 2H), 4.69 (s, H), 4.49 (s, H), 1.88 (s, 3H ); ^13^C NMR (CD_3_CN): δ 171.37, 97.90, 78.75, 75.35,70.51, 69.03, 49.56, 22.76 ppm; IR (cm^−1^) ṽ = 3297, 2991, 1651, 1638, 1533, 1283, 1258, 1155, 1133, 1022, 953, 890, 824, 749, 677, 638, 615, 597, 532, 493, 471; elemental analysis for C_8_H_11_N_5_O_14_ (401.20): calcd C 23.95, H 2.76, N 17.46%; found: C 24.04, H 2.72, N 17.38%.

## 5. Conclusions

A new energetic compound, (3R,4R,6R)-3-acetamido-6-((nitrooxy)methyl)tetrahydro-2H-pyran-2,4,5-triyl trinitrate (**1**), was synthesized and fully characterized. This compound was prepared with an excellent yield of 95% by nitrating N-Acetyl-D-Glucosamine. N-Acetyl-D-Glucosamine is commercially available and can be obtained via the simple hydrolysis of chitin biopolymer, meaning that Compound **1** is an attractive material for various applications. The structure of Compound **1** was determined through single-crystal X-ray diffraction. The obtained results confirm that Compound **1** has high nitrogen contents and better density in comparison to NC and MCNC. In addition, Compound **1** was calculated to exhibit better detonation performance than NC and MCNC, as well as a comparable specific impulse. Compound **1** exhibits lower sensitivity to impact in comparison to NC and MCNC. The potential for Compound **1** to be used as propellant component was evaluated by replacing nitrocellulose in typical propellant compositions. These properties make Compound **1** attractive for potential integration in a broad range of energetic formulations.

## Figures and Tables

**Table 1:** Physical properties, sensitivity data, and calculated detonation properties of Compound **1**.

	Compound 1	NC [29]	MCNC [29]
Experimental formula	C_8_H_11_N_5_O_14_	C_6.01_H_7.10_N_2.63_O_10.90_	C_5.96_H_7.15_N_2.75_O_10.94_
Molecular mass (g/mol)	401.20	290.44	292.21
T_d_ (°C) ^a^	154	194	192
Impact sensitivity (J) ^b^	15	3.2	2.8
Friction sensitivity (N) ^c^	128	355	350
N (%) ^d^	17.46	12.68	13.17
Ω (%) ^e^	−29.91	−25.73	−24.94
ρ (g/cm^−3^ ) ^f^	1.721	1.673	1.691
Δ_f_H_m_ (kJ/mol) ^g^	−785.8	−714	−573
P (GPa) ^h^	25.6	23	25
V_d_ (m/s) ^i^	7717	7456	7683
Isp (s) ^j^	240	239	250

Legend: ^a^ decomposition temperature (onset); ^b^ BAM drop hammer tester; ^c^ BAM friction tester; ^d^ nitrogen content; ^e^ oxygen balance; ^f^ density measured by using a gas pycnometer at 25 °C; ^g^ molar enthalpy of formation; ^h^ detonation pressure calculated using EXPLO5 V6.05; ^i^ detonation velocity calculated using EXPLO5 V6.05; ^j^ specific impulse.

**Table 2:** Compositions of typical propellant formulations and formulations with nitrocellulose replaced with Compound **1**.

Propellant	Composition	Percentage of Total (%)	I_sp_ ( s) ^a^
JA2	Nitrocellulose (NC) (13.04% N)	59.5	243
	Nitroglycerine (NG)	14.9	
	Diethylene glycol dinitrate	24.8	
	Ethyl centralite (EC)	0.7	
	Magnesium oxide	0.05	
	Graphite	0.05	
F1	Compound **1**	59.5	246
	Nitroglycerine (NG)	14.9	
	Diethylene glycol dinitrate	24.8	
	Ethyl centralite (EC)	0.7	
	Magnesium oxide	0.05	
	Graphite	0.05	
M30	Nitrocellulose (NC) (12.68% N)	28	228
	Nitroglycerine (NG)	22.5	
	Nitroguanidine (NQ)	47.7	
	Ethyl centralite (EC)	1.5	
	Cryolite	0.3%	
F2	Compound **1**	28	232
	Nitroglycerine (NG)	22.5	
	Nitroguanidine (NQ)	47.7	
	Ethyl centralite (EC)	1.5	
	Cryolite	0.3	
M9	Nitrocellulose (NC) (14% N)	57.75	254
	Nitroglycerine (NG)	40.00	
	Ethyl centralite (EC)	0.75	
	Potassium nitrate	1.50	
F3	Compound **1**	57.75	254
	Nitroglycerine (NG)	40.00	
	Ethyl centralite (EC)	0.75	
	Potassium nitrate	1.50	

^a^ Specific Impulse calculated using EXPLO5 V 6.05.

## Data Availability

Data are contained within the article and the Supplementary Materials.

## Supplementary Materials

The following are available online, Table S1: Crystallographic data for compound **1**, Table S2: Calculated (B3LYP/6-31+G**// M062X/def2QZVPP) total energy (E0), zero-point energy (ZPE), values of the correction (H_T_), and heats of formation (HOF) for **1**, Figure S1, S2: ^1^H NMR and ^13^C NMR spectrum of compound **1** in CD_3_CN, Figure S3: DSC curve for compound **1**, Scheme S1: Isodesmic reaction for calculating heat of formation for compound **1**.

## Sample Availability

Samples of the compounds are available from the authors.
