# Supplementary material for: Nitration of Chitin Monomer: From Glucosamine to Energetic Compound
Source: Molecules. 2021 Dec 12;26(24):7531. doi: 10.3390/molecules26247531 (PMC8704513; doi:10.3390/molecules26247531)
Supplement: Supplementary file 1 [file molecules-26-07531-s001.zip › molecules-1511243-supplementary.pdf]

Supporting Information

Nitration of Chitin Monomer:
From Glucosamine to Energetic Compound

Hui Dou [1], Yuxuan Zheng [2], Manyi Qu [3], Peng Chen [1], Chunlin He [1,2,4,5*], Michael Gozin [6,7,8*] and Siping Pang [1,2*]

1 Experimental Center of Advanced Materials, School of Materials Science and Engineering, Beijing Institute of Technology, Beijing 100081, China; douhui@bit.edu.cn (H.D.); pengchen@bit.edu.cn (P.C.)
2 State Key Laboratory of Explosion Science and Technology, Beijing Institute of Technology, Beijing 100081, China; yxzheng@bit.edu.cn (Y. Z.)
3 Shanxi North Xingan Chemical Industry CO.LTD，Taiyuan 030008; China. qumanyi2005@163.com (M. Q.)
4 Yangtze Delta Region Academy of Beijing Institute of Technology, Jiaxing 314019, China.
5 Chongqing Innovation Center, Beijing Institute of Technology, Chongqing 401120, China.
6 School of Chemistry, Faculty of Exact Science, Tel Aviv University, Tel Aviv 69978, Israel.
7 Center for Nanoscience and Nanotechnology, Tel Aviv University, Tel Aviv 6998701, Israel.
8 Center for Advanced Combustion Science, Tel Aviv University, Tel Aviv 6997801, Israel.

* Correspondence: chunlinhe@bit.edu.cn (C.H.); cogozin@gmail.com (M.G.); pangsp@bit.edu.cn (S.P.).

1. X-ray diffraction data S2
2. ^{1}H , ^{13}C NMR Spectra and DSC curve...S3
3. Gaussian Calculations S4

1. X-ray diffraction data.

Table S1. Crystallographic data for compound **1.**

**Empirical formula**	$\mathbf{C_8H_{11}N_5O_{14}}$
Formula weight	401.22
Temperature/K	296(2)
Crystal system	Triclinic
Space group	P1
a/Å	5.093(15)
b/Å	8.63(3)
c/Å	17.69(5)
α/°	95.35(4)
β/°	90.88(4)
γ/°	90.23(4)
Volume/$Å^3$	774(4)
Z	1
ρ g/cm^3	1.722
μ/mm^{-1}	0.169
F(000)	412.0
Crystal size/mm^3	0.15 × 0.10 × 0.06
Radiation	MoKα (λ = 0.71073)
2Θ range for data collection/°	4.626 to 50.496
Index ranges	-6 ≤ h ≤ 6, -10 ≤ k ≤ 10, -21 ≤ l ≤ 21
Reflections collected	6048
Independent reflections	5079 [Rint = 0.0932, Rsigma = 0.3267]
CCDC	2104267

2 ^1H , ^{13}C NMR spectra and DSC curve for compound **1.**

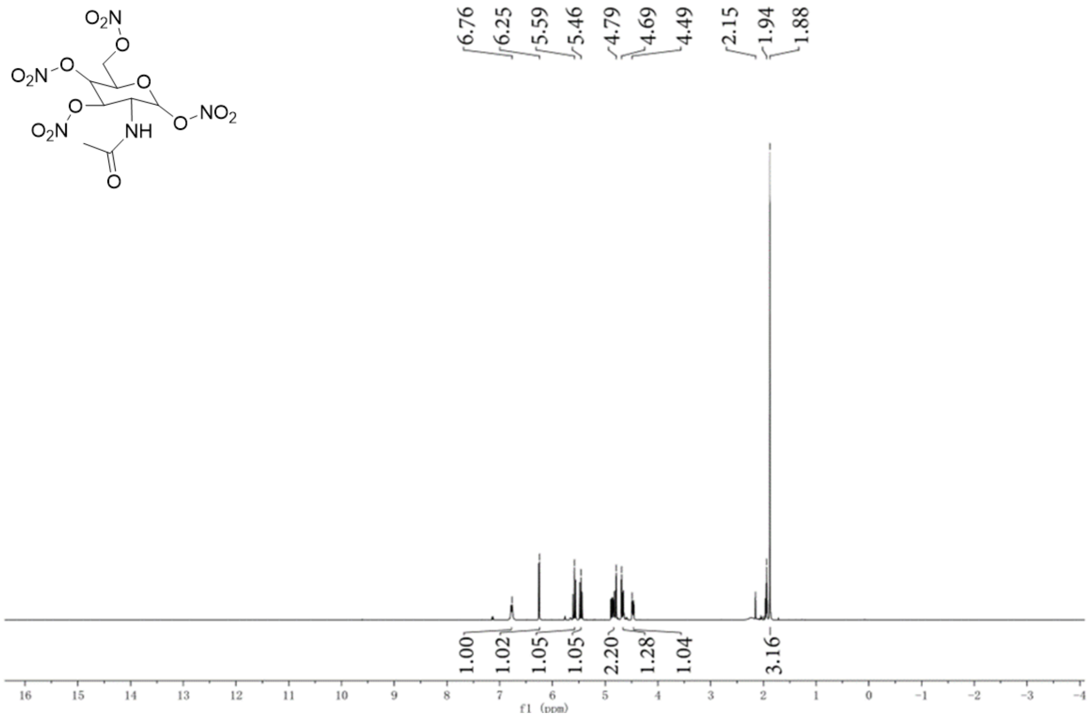

Figure S1. ^1H NMR spectrum of compound **1** in CD_3CN.

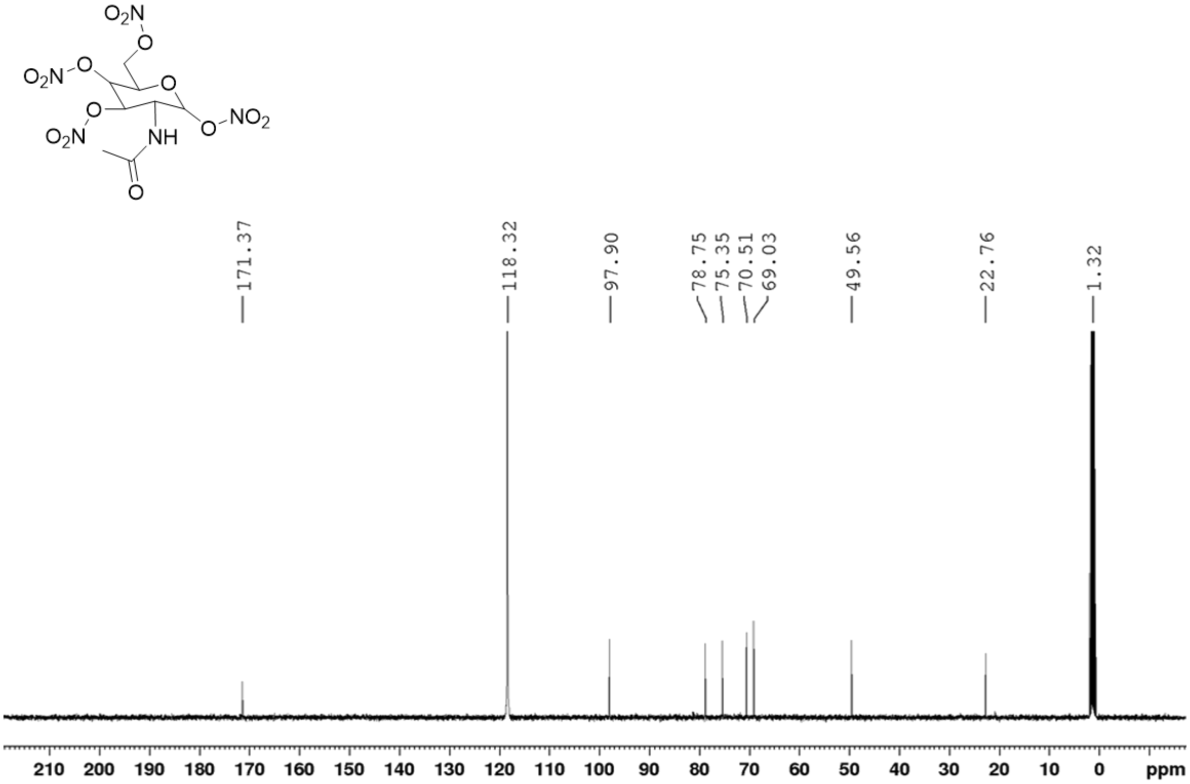

Figure S2. ^{13}C NMR spectrum of compound **1** in CD_3CN.

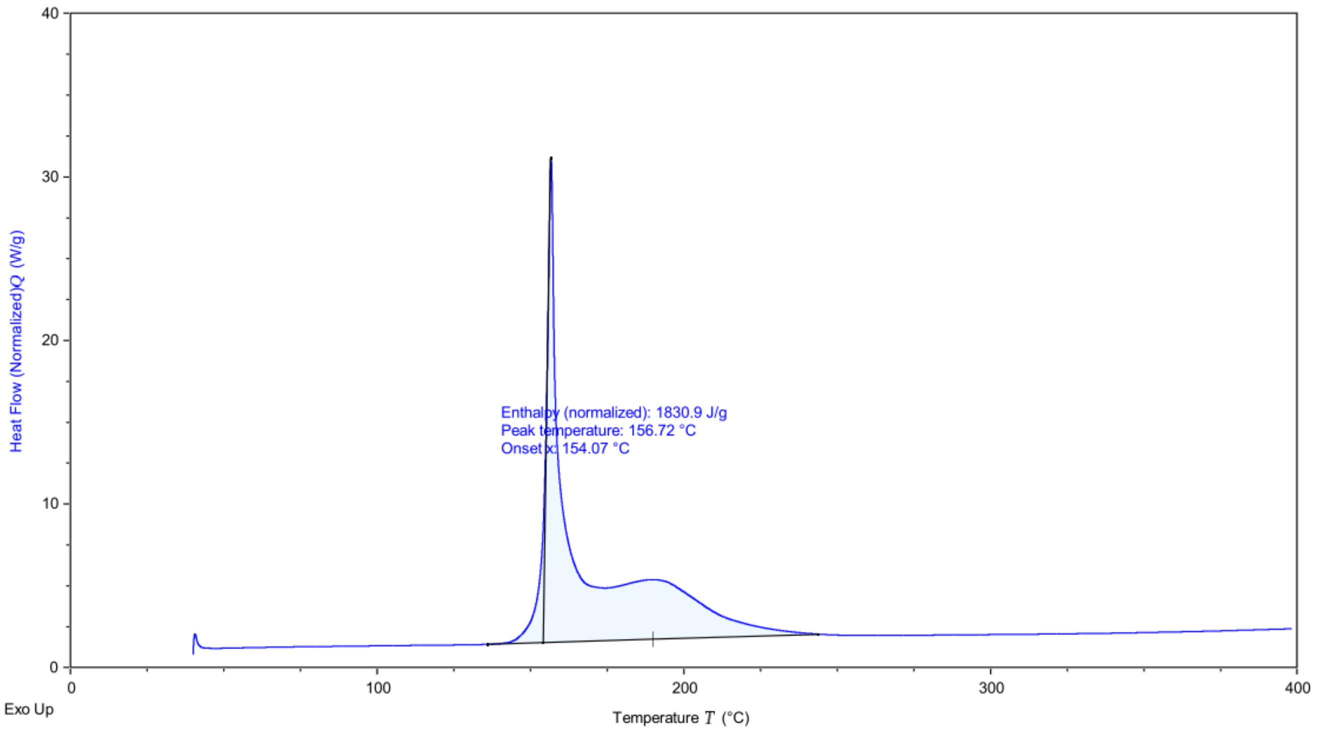

Figure S3 DSC curve for compound **1**.

3. Gaussian Calculations

All quantum chemical calculations were carried out using the Gaussian 09 program package and visualized by GaussView 5.0. The geometric optimization and frequency analyses of the structures were carried out using the B3LYP functional with 6–31+G(d) basis set, and single energy points were calculated at the M062X/def2QZVPP level. All of the optimized structures were characterized to be true local energy minima on the potential energy surface without imaginary frequencies. The heat of formation was determined using an isodesmic reaction (Figure S3). All of the heats of formation were obtained from the NIST WebBook. The heat of sublimation was calculated according to an empirical formulation based the melting point or decomposition temperature. ΔH_{sub} = 0.188*(T_{dec}+273)=0.188*(154+273)= 80.3 kJ mol^{-1}.

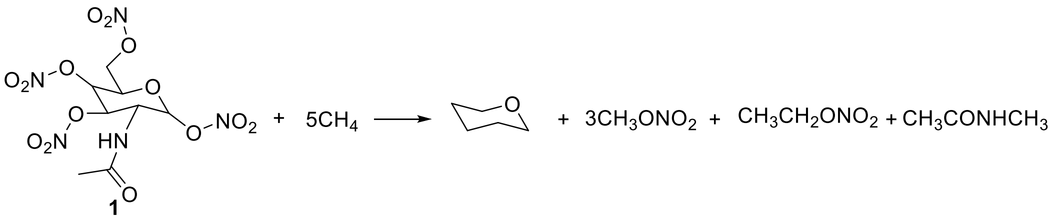

Scheme S1. Isodesmic reaction for calculating heat of formation for compound **1**.

Table S2. Calculated (B3LYP/6-31+G**// M062X/def2QZVPP) total energy (E0), zero-point energy (ZPE), values of the correction (H_T), and heats of formation (HOF) for **1**.

**Compound**	**ZPE**	$\mathbf{H_T}$	$\mathbf{E_0}$	**Corrected** $\mathbf{E_0}$	$\mathbf{\Delta H_{f\ gas}}$**(kJ mol**$^{-1}$**)**	$\mathbf{\Delta H_{sub}}$	$\mathbf{\Delta H_{f\ solid}}$
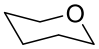	0.146956	0.153327	-271.7714738	-271.62403	-223.8 [a]		
CH_3ONO_2	0.054504	0.060419	-320.2182942	-320.16006	-122 [a]		
$CH_3CH_2ONO_2$	0.082991	0.090139	-359.5336884	-359.44687	-155 [a]		
$CH_3CONHCH_3$	0.102159	0.109837	-248.5335705	-248.42782	-248 [a]		
CH_4	0.045014	0.048823	-40.5062133	-40.45919	-74.6 [a]		
Compound **1**	0.255546	0.282736	-1637.992806	-1637.72029	-705.5	80.3	-785.8

[a] Data from NIST WebBook.
